# Assessing Esthetic and Functional Benefits of Three Types of Maxillary Partial Denture Designs over Five Years

**DOI:** 10.3390/dj13120610

**Published:** 2025-12-18

**Authors:** Sanja Peršić Kiršić, Asja Čelebić, Irina Filipović-Zore, Ljiljana Strajnić, Nikola Petričević

**Affiliations:** 1 Department of Removable Prosthodontics, School of Dental Medicine, University of Zagreb, Gundulićeva 5, 10000 Zagreb, Croatia; celebic@sfzg.hr (A.Č.); petricevic@sfzg.hr (N.P.); 2 Department of Oral Surgery, School of Dental Medicine, University of Zagreb, Gundulićeva 5, 10000 Zagreb, Croatia; filipovic@sfzg.hr; 3 Clinical Hospital Centre, 10000 Zagreb, Croatia; 4 Clinic for Dentistry of Vojvodina, Faculty of Medicine, University of Novi Sad, 21000 Novi Sad, Serbia; lilistrajnic@gmail.com

**Keywords:** removable partial denture, maxilla, esthetics, OHRQoL, chewing function, d-PROMs, mini-implant-retained RPD, clasp-retained RPD, attachment-retained RPD

## Abstract

**Background/Objectives:** The design of removable partial dentures (RPDs) influences long-term clinical success and patient satisfaction. Maxillary Kennedy Class I partial edentulism can be treated with clasp-retained (C-RPD), attachment-retained (A-RPD), or implant-retained (I-RPD) removable partial dentures. Evidence on their long-term effects on esthetics, oral health-related quality of life (OHRQoL), and masticatory function is limited. This study compared patient-reported outcomes of these three RPD types over five years. **Methods:** Eighty-eight patients received C-RPD, semi-precision attachment A-RPD, or mini-implant I-RPD. Outcomes: Esthetic satisfaction (OES), OHRQoL (OHIP-14), and chewing function (CFQ) were assessed pre-treatment, post-treatment, and at 1- and 5-year follow-ups. Treatment effect sizes were analyzed using ANCOVA adjusting for baseline scores, age, gender, and education, while long-term trends were assessed by repeated-measures ANCOVA. **Results:** Treatment group significantly influenced outcomes. C-RPD users reported lower esthetic satisfaction, OHRQoL, and chewing function than A-RPD or I-RPD users (*p* < 0.001). Baseline scores predicted post-treatment outcomes (lower pre-treatment = lower post-treatment scores). Over five years, OES worsened in all groups (*p* = 0.004) with C-RPDs, consistently showing the worst scores. OHIP-14 scores increased most in C-RPD wearers (17.6 → 28.4; *p* < 0.001) indicting worst OHRQoL, while A-RPD and I-RPD scores remained significantly lower (10.8 → 17.4 and 10.9 → 13.1, respectively). CFQ scores followed similar trend: C-RPD: 20.43; A-RPD: 13.59; I-RPD: 12.40 (*p* < 0.001). Age, gender, and education had minimal or no significant impact. **Conclusions**: C-RPDs are associated with lower esthetic satisfaction, poorer OHRQoL and reduced chewing function, with a marked decline over five years. In contrast A-RPDs and I-RPDs showed higher treatment effect sizes and more stable patient-reported outcomes over 5 years. Due to study limitations, results should be interpreted cautiously, as they may reflect treatment self-selection rather than prosthesis design alone.

## 1. Introduction

Orofacial esthetics has become a central focus of contemporary dentistry and is recognized as one of the four key dimensions of oral health-related quality of life (OHRQoL), alongside oral function, comfort/pain, and psychosocial well-being [1,2]. In addition to restoring masticatory performance, patients increasingly evaluate dental treatments according to their ability to enhance facial appearance and smile esthetics, both of which represent crucial determinants of perceived treatment success [3,4,5,6,7,8,9,10,11,12]. Dentofacial appearance influences not only judgments of facial attractiveness but also perceptions of personality and psychosocial adjustment, thereby contributing to self-confidence and overall quality of life [13,14,15,16,17,18,19,20,21,22,23].

Demographic trends indicate that the need for removable partial dentures (RPDs) will continue to grow as populations age and life expectancy increases [24,25]. For partially edentulous individuals, particularly those requiring maxillary RPDs, both functional and esthetic outcomes strongly influence treatment acceptance and satisfaction [6,11,16,17,18,25,26,27,28,29,30,31,32,33,34,35]. In this context, dental patient-reported outcome measures (d-PROMs) offer essential insight into how oral health conditions and prosthodontic therapy affect daily functioning beyond traditional clinical assessments. These measures allow evaluation of treatment effectiveness, patient satisfaction, and long-term adaptation, while also supporting communication in shared decision-making and informing cost-effectiveness analyses [11,36].

Among d-PROMs, the Oral Health Impact Profile (OHIP), particularly the OHIP-14 and OHIP-5, remains one of the most widely used instruments for assessing OHRQoL due to strong cross-cultural validation and availability in multiple languages. The Orofacial Esthetic Scale (OES) provides a validated unidimensional assessment of patients’ perceptions of orofacial esthetics [11,37,38], and the Chewing Function Questionnaire (CFQ) measures self-reported masticatory ability [39]. Together, these instruments enable a comprehensive evaluation of both esthetic and functional outcomes in prosthodontic rehabilitation [1,2,37,38,39]. However, the interaction between esthetic appearance and chewing function in patients rehabilitated with RPDs is not yet fully clarified.

Different maxillary RPD designs offer distinct advantages and limitations. Conventional clasp-retained RPDs (C-RPDs) are widely used and economically accessible but may compromise esthetics due to visible metal clasps [17,40,41]. Extracoronal attachment-retained RPDs (A-RPDs) reduce the visibility of metal components and improve esthetic outcomes, while mini-implant-retained RPDs (I-RPDs) provide additional benefits in retention, stability, function, and appearance [6,17,40,41,42,43,44,45,46,47,48]. Nevertheless, robust long-term comparative evidence examining both esthetic and functional dimensions across these RPD types remains limited.

Therefore, the aim of this study was to evaluate the five-year outcomes of three maxillary RPD designs—clasp-retained (C-RPD), extracoronal attachment-retained (A-RPD), and mini-implant-retained (I-RPD) by comparing their effects on orofacial esthetics, masticatory function, and OHRQoL. In addition, the effect size of treatment outcomes in relation to prosthesis type was analyzed to help identify the rehabilitation option offering the most favorable balance of functional and esthetic benefits.

## 2. Materials and Methods

### 2.1. Study Design

This longitudinal, observational, and comparative study included three groups of participants categorized according to the type of maxillary removable partial denture (RPD) provided: clasp-retained (C-RPD), extracoronal precision attachment-retained (A-RPD), and mini-implant-retained (I-RPD). All patients exhibited Kennedy Class I maxillary partial edentulism (absence of premolars and molars), with only four to six anterior teeth left (canines were terminal teeth). Patients have been referred to the Department of Prosthodontics for the fabrication of new maxillary RPDs. The remaining anterior teeth demonstrated a favorable prognosis: adequate bone support (>50% of root length surrounded by bone; crown-to-root ratio approximately 1:>1), absence of pathologic mobility, no active periodontal disease, vital or successfully endodontically treated teeth, no root resorption, sufficient coronal tooth structure, and acceptable oral hygiene. In the A-RPD group, metal–ceramic fixed partial dentures (FPDs) incorporating extracoronal attachments were fabricated. Four to six remining teeth were abutments of that bridge with canines being terminal abutments. In contrast, participants in the C-RPD and I-RPD groups retained their natural anterior teeth, which were restored with individual crowns, short-span fixed bridges, or fillings, when necessary.

Eligibility criteria for all participants to be included in the study were buccolingual ridge width between 4.0 and 5.9 mm in the premolar region, and alveolar bone height ≥12 mm. Participants with ridge width > 6.0 mm were excluded, as they were suitable candidates for standard-width implants. According to the Cawood and Howell classification [49], all participants exhibited posterior maxillary and mandibular ridge morphology between Types III and IV. Although the ridges were narrow and reduced in width, none were fully knife-edged, which would have contraindicated flapless mini-implant placement in the I-RPD group. However, the ridge dimensions were insufficient for the placement of standard-width implants, which is why mini-implants were indicated in eligible participants.

All participants had previously worn maxillary and mandibular clasp-retained RPDs, which contributed to reduced residual ridge volume, in some cases further affected by sinus pneumatization following molar extraction. As a result, they were not suitable candidates for implant-supported fixed prostheses without additional demanding augmentation procedures. In the opposing (mandibular) arch, all participants also exhibited Kennedy Class I partial edentulism and were rehabilitated with conventional removable partial dentures.

Following clinical examination and panoramic radiography and/or CBCT evaluation, all participants were presented with three treatment options (C-RPD, A-RPD, I-RPD), including detailed explanation of treatment sequence, expected functional and esthetic outcomes, maintenance needs, duration, and cost considerations. Mini-implants were provided free of charge as part of a research grant (Project No. IP-2014-09-1218, Croatian Science Foundation). The study was non-randomized, and treatment allocation was based on patient preference following a comprehensive explanation. Each participant made an informed decision regarding the preferred prosthetic modality and provided written informed consent prior to enrollment.

The study was conducted in accordance with the Declaration of Helsinki and was approved by the Institutional Ethics Committee of the University of Zagreb, School of Dental Medicine (Ethical Approval No.: 05-PA-26-6/2015; approval date: 16 April 2015). The study was conducted between December 2015 and February 2024, with a five-year follow-up after prosthesis delivery.

### 2.2. Sample Size

The sample size was determined based on previous studies [6,17,42,50], using a significance level of *p* < 0.05, 80% statistical power, and an anticipated 30% dropout rate over the five-year follow-up period. The minimum required sample size was calculated to be 28 participants per group at baseline.

To enhance statistical power, a slightly larger initial cohort was recruited, resulting in 105 participants (37 C-RPD, 36 A-RPD, 32 I-RPD). Only participants who completed the full five-year follow-up were included in the final analysis, yielding 88 participants (30 C-RPD, 31 A-RPD, 27 I-RPD). During the first year, two participants (6.5%) discontinued participation from both the C-RPD and A-RPD groups, while one participant (3.1%) dropped out of the I-RPD group. Over the subsequent years, an additional five participants withdrew from the C-RPD group, three from the A-RPD group, and four from the I-RPD group.

### 2.3. Participants and Procedures

All prosthetic procedures, as well as surgical procedures in the I-RPD group, were carried out by prosthodontic and oral surgery residents under the supervision of experienced specialists, following a standardized clinical protocol. All participants received new maxillary RPDs. In the mandibular arch, Kennedy Class I partial edentulism was treated with new mandibular RPDs unless an existing conventional RPD (not older than two years) demonstrated satisfactory functional and esthetic performance, in which case it was retained. All mandibular RPDs were constructed with cast cobalt–chromium (CoCr) frameworks incorporated in acrylic resin. A lingualized occlusion and non-balanced posterior teeth set-up were used for all RPDs. Canine guidance was established when feasible; otherwise, group function was used. Bilateral balanced occlusion was intentionally avoided, as it is not indicated for RPDs with tooth or implant abutments.

Exclusion criteria for receiving new RPDs included mental illness preventing proper prosthesis use, inability to maintain oral hygiene or handle the prosthesis due to frailty or motor impairment, and systemic conditions contraindicating RPD therapy. Patients who smoked up to 20 cigarettes per day and those with well-controlled diabetes were not excluded. In the I-RPD group, additional eligibility criteria followed standard implant therapy guidelines [50,51]. Although all patients had sufficient ridge dimensions in the premolar region to allow the placement of narrow-diameter implants, mini-implant (MDI) insertion was performed only in those who consented to receiving implant-assisted retention of the maxillary RPD.

In the I-RPD group, MDIs were inserted flaplessly, one or two tooth positions distal to the last remaining canine, following the planned insertion path of the RPD. Mini-implants were early loaded (6–8 weeks post-insertion) when the insertion torque was ≥35 Ncm; otherwise, loading was delayed for three to four months. Detailed descriptions of the MDI placement protocol and corresponding prosthetic procedures have been reported in previous publications [6,42,50].

Patients in the A-RPD group received metal–ceramic anterior fixed partial dentures incorporating extracoronal male components (patrices), while the corresponding female components (matrices) were integrated into the RPD framework (Ceka Preci-Sagix, Waregem, Belgium). This semi-precision attachment system allowed controlled denture movement, specifically vertical displacement and limited posterior rotation, thereby enhancing retention and esthetics while reducing visible clasping.

All maxillary dentures were fabricated with cast Co–Cr frameworks featuring a U-shaped major connector and cingulum rests placed on prepared anterior teeth or crown rest seats. C-RPDs were retained with wrought-wire clasps (preferred over cast metal clasps for esthetic reasons) used in combination with rigid reciprocal components and/or indirect retainers such as cingulum rests. In A-RPDs, comparable framework elements were incorporated; however, extracoronal semi-precision attachments were used in place of visible clasps. In the I-RPD group, cingulum rests were also included, and retention was achieved through metal housings containing rubber O-rings embedded within the acrylic denture base.

### 2.4. Dental Patient Reported Outcome Measures (d-PROMS)

Three validated dental patient-reported outcome measures (d-PROMs) were used in this study. Patients’ self-perceived orofacial esthetics was assessed using the Croatian version of the Orofacial Esthetic Scale (OES-CRO), which consists of eight items [52]. All questionnaires were primarily self-administered in a quiet clinical environment, without interviewer involvement, to minimize social desirability and interviewer bias. However, when a participant required clarification of a specific item, a trained researcher provided standardized explanation without influencing or suggesting any particular response.

Participants rated their self-perceived orofacial esthetics on a 1–5 Likert scale (1 = completely dissatisfied; 5 = completely satisfied), resulting in a total OES score ranging from 8 to 40, with higher scores indicating greater satisfaction.

Chewing function was assessed using the Chewing Function Questionnaire (CFQ), which consists of 10 items scored on a 0–4 Likert scale, where higher scores reflect greater chewing difficulty (i.e., poorer masticatory function). The CFQ summary score therefore ranged from 0 to 40, with higher scores indicating more chewing problems [39].

Finally, oral health-related quality of life (OHRQoL) was assessed using the Croatian adaptation of the OHIP-14 questionnaire. Each item was rated on a 0–4 Likert scale, yielding total scores from 0 to 56, where higher scores indicate greater impairment and thus poorer OHRQoL [53].

All three instruments have been validated in Croatian populations and have demonstrated excellent reliability and construct validity [39,52,53]. At each assessment point, participants rated their experiences over the previous seven days [54].

Pre-treatment d-PROMs were collected during the initial clinical examination (pre-treatment scores). Post-treatment assessments were conducted one to two months after new RPD delivery (and after implant loading in the I-RPD group) to allow sufficient time for denture adjustments and functional adaptation. The assessments were also performed at the 1-year and 5-year follow-up visits (post-treatment scores).

### 2.5. Statistical Analysis

Statistical analyses were performed using SPSS software, version 20 (IBM Corp., Armonk, NY, USA). Descriptive statistics and the one-sample Kolmogorov–Smirnov test were used for preliminary analyses. To estimate treatment effect sizes, analysis of covariance (ANCOVA, General Linear Model) was conducted with post-treatment scores as the dependent variable, treatment groups (C-RPD, A-RPD, I-RPD) and gender as fixed factors, and baseline scores, education level, and age as covariates. Effect sizes were interpreted based on partial eta squared (ηp^2^): a small effect was defined as ηp^2^ = 0.01 (explaining 1% of the variance), a medium effect as ηp^2^ = 0.06 (explaining 6% of the variance), and a large effect as ηp^2^ ≥ 0.14 (explaining 14% or more of the variance) [55]. Post hoc pairwise comparisons between treatment groups were performed using the Bonferroni test.

Longitudinal changes were analyzed using repeated-measures ANCOVA with time (1 = post-treatment, 2 = 1 year, 3 = 5 years) as the within-subjects factor, treatment group and gender as between-subjects factors, and education and age as covariates.

## 3. Results

Of the 105 participants initially enrolled, 88 completed the 5-year follow-up (30 C-RPD, 31 A-RPD, 27 I-RPD), including 34 men and 54 women. Participants were 43–80 years old (mean age = 63.93 ± 8.04 years) and varied in educational background: 14 had completed primary school, 13 vocational school, 24 secondary school, 25 held a bachelor’s degree, and 12 a master’s degree or higher.

At baseline, 78 participants (88.6%) received new mandibular RPDs, while 10 (11.4%) retained well-functioning existing mandibular RPDs not older than two years. One mandibular RPD was relined prior to maxillary prosthesis fabrication. The distribution of retained mandibular RPDs cross groups was as follows: C-RPD = 4, A-RPD = 3, and I-RPD = 3. During the 5-year follow-up, no mandibular or maxillary RPDs were replaced; however, routine maintenance was performed when needed.

In the I-RPD group (*n* = 27), mini-implants were early loaded in 17 participants (insertion torque ≥ 35 Ncm), while delayed loading was applied in 10 participants following a 3–4-month healing period.

Throughout the observed period of five years periodontal health of the anterior abutment teeth remained clinically acceptable in all three groups, with no evidence of progressive attachment loss or increased tooth mobility. In the I-RPD group, no mini-implant failures occurred, and no clinically relevant marginal bone loss was detected, resulting in a 100% mini-implant survival rate. Soft-tissue conditions around the MDIs and beneath the denture bases remained acceptable, and no cases of peri-implant pathology requiring intervention were recorded.

Mean values, standard deviations, and score ranges for the OES, OHIP-14, and CFQ at pre-treatment, post-treatment, 1-year, and 5-year follow-up are presented in Table 1. for all three treatment groups (C-RPD, A-RPD, I-RPD).

### 3.1. Esthetic Outcomes (OES)

A univariate ANCOVA demonstrated a significant effect of maxillary RPD type on post-treatment OES scores after adjusting for pre-treatment values, gender, age, and education (F (8,79) = 17.128, *p* < 0.001), explaining 63% of the variance (R^2^ = 0.634; adjusted R^2^ = 0.597). RPD type showed a large effect size (partial η^2^ = 0.577, *p* < 0.001), indicating substantial differences in esthetic outcomes between treatment groups. Baseline (pre-treatment) OES scores were also a significant predictor (partial η^2^ = 0.140, *p* = 0.001), with higher pre-treatment scores associated with higher post-treatment scores. Age, education, and gender did not significantly influence post-treatment OES outcomes (partial η^2^ ≤ 0.037).

Post hoc comparisons revealed significantly lower post-treatment OES scores in the C-RPD group compared with both the A-RPD and I-RPD groups (*p* < 0.05). No significant differences were found between the A-RPD and I-RPD groups, both of which demonstrated superior esthetic outcomes compared with the C-RPD group.

**Figure 1:**
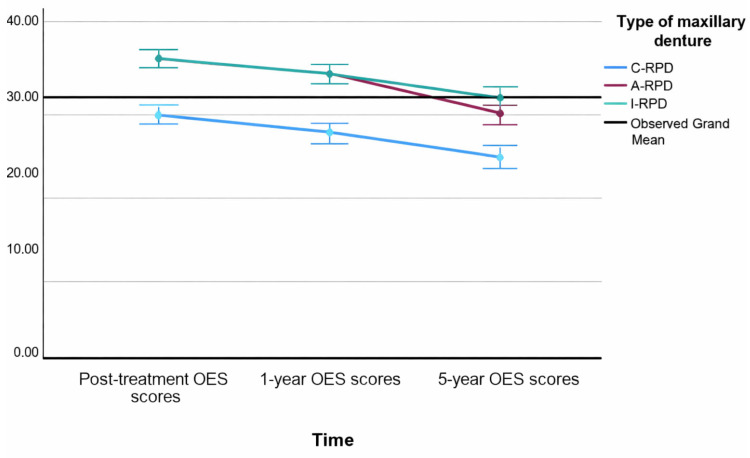
Estimated marginal means with 95% Confidence Intervals of the OES summary scores over time by types of maxillary RPDs.

Repeated-measures ANCOVA assessing longitudinal change in OES scores demonstrated a statistically significant effect of time (F (1.39, 111.2) = 6.18, *p* = 0.008, Greenhouse–Geisser corrected), indicating a gradual decrease in esthetic satisfaction over the five-year period. A significant linear trend was observed from post-treatment to the five-year follow-up (F (1, 80) = 8.69, *p* = 0.004). Estimated marginal means showed that OES scores declined from 34.4 (SD = 4.0) immediately after treatment to 32.6 (SD = 4.3) at the one-year follow-up, and to 29.4 (SD = 4.5) at the five-year follow-up (Figure 1).

The effect of treatment group was highly significant (F (2, 80) = 43.25, *p* < 0.001). Patients rehabilitated with C-RPDs consistently reported the lowest esthetic satisfaction across all time points (mean = 27.8; 95% CI: 26.7–28.8), whereas those with A-RPDs and I-RPDs reported significantly higher satisfaction (A-RPD: mean = 33.8; 95% CI: 32.4–35.3; I-RPD: mean = 34.6; 95% CI: 33.5–35.8). No statistically significant differences were observed between the A-RPD and I-RPD groups, although the I-RPD group demonstrated slightly higher mean scores.

Age, gender, and education did not significantly influence OES outcomes (*p* > 0.05). Figure 1 illustrates the estimated marginal means with 95% confidence intervals for OES scores over time for each treatment group.

### 3.2. Oral Health-Related Quality of Life (OHIP-14)

The univariate ANCOVA for post-treatment OHIP-14 scores, adjusted for pre-treatment values, age, education, and gender, demonstrated a significant effect of treatment group (F (2,79) = 43.15, *p* < 0.001, partial η^2^ = 0.522), indicating a large effect size and substantial variation in OHRQoL outcomes depending on the type of maxillary RPD. The overall model was highly significant (F (8,79) = 16.61, *p* < 0.001), explaining 63% of the variance in post-treatment OHIP-14 scores (R^2^ = 0.627; adjusted R^2^ = 0.589). Pre-treatment OHIP-14 scores were a strong predictor of post-treatment scores (F (1,79) = 19.73, *p* < 0.001, partial η^2^ = 0.200). Age showed a small but significant effect (F (1,79) = 4.57, *p* = 0.036, partial η^2^ = 0.055), with older participants reporting slightly higher (worse) OHIP-14 scores. Education and gender did not significantly influence outcomes (*p* > 0.20).

Adjusted mean post-treatment scores indicated that patients rehabilitated with C-RPDs reported significantly worse OHRQoL (mean = 17.7; 95% CI: 16.5–18.9) compared with those rehabilitated with A-RPDs (mean = 10.6; 95% CI: 8.9–12.2) and I-RPDs (mean = 10.3; 95% CI: 9.0–11.5). Bonferroni-adjusted pairwise comparisons confirmed that the C-RPD group had significantly higher (worse) OHIP-14 scores than both the A-RPD (mean difference = 7.17, *p* < 0.001) and I-RPD groups (mean difference = 7.48, *p* < 0.001), whereas no significant difference was observed between the A-RPD and I-RPD groups (*p* = 1.000).

Longitudinal analysis (ANCOVA) demonstrated significant changes in OHIP-14 scores over time (F (1.86,148.9) = 4.06, *p* = 0.021, partial η^2^ = 0.093). Scores increased from the post-treatment assessment to the 1-year and 5-year follow-ups in all groups; however, the extent of change differed substantially by treatment type. The interaction between time and treatment group was significant (F (2,80) = 10.51, *p* < 0.001, partial η^2^ = 0.361), indicating that maxillary RPD design strongly influenced the trajectory of OHRQoL over the five-year period.

Scores increased from post-treatment (C-RPD: 17.6, A-RPD: 10.8, I-RPD: 10.85) to 1-year follow-up (C-RPD: 18.8, A-RPD: 11.45, I-RPD: 11.33), and further at 5 years (C-RPD: 28.43, A-RPD: 17.35, I-RPD: 13.11). The interaction between time and treatment type was significant (F = 10.51, *p* < 0.001, partial η^2^ = 0.361), indicating that patients with C-RPDs experienced the most pronounced deterioration, whereas those with attachment-retained and implant-supported dentures maintained stable OHRQoL over time.

Between-subjects analysis confirmed a strong overall effect of denture type on OHIP-14 scores (F (2,80) = 69.38, *p* < 0.001, partial η^2^ = 0.634). Post hoc comparisons indicated significantly higher (worse) OHIP-14 scores in the C-RPD group compared with both the A-RPD (mean difference = 9.31, *p* < 0.001) and I-RPD groups (mean difference = 10.81, *p* < 0.001), while no significant difference was found between the A-RPD and I-RPD groups (*p* > 0.05).

**Figure 2:**
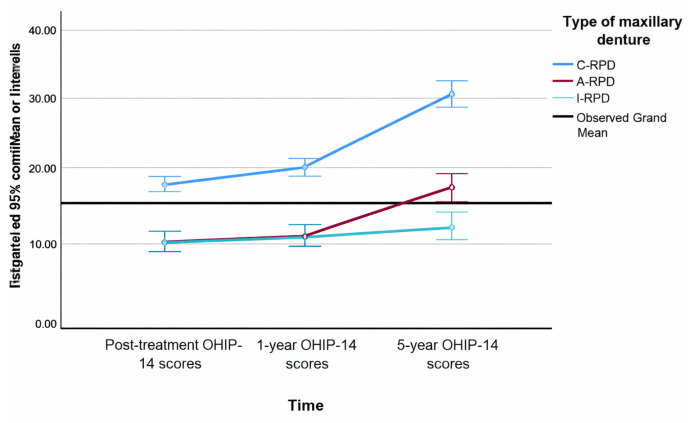
Estimated marginal means with 95% Confidence Intervals of the OHIP-14 summary scores over time by types of maxillary RPDs.

These results indicate that attachment-retained and implant-retained RPDs provide substantially better and more stable OHRQoL outcomes than conventional clasp-retained RPDs across a five-year period. Estimated marginal means with 95% confidence intervals (CI) for OHIP-14 scores over time by treatment group are shown in Figure 2.

### 3.3. Chewing Function (CFQ)

A univariate ANCOVA demonstrated a significant effect of maxillary RPD type on post-treatment CFQ scores after adjusting for baseline CFQ, age, gender, and education (F (8,79) = 9.07, *p* < 0.001), explaining 48% of the variance in post-treatment chewing function (R^2^ = 0.479, adjusted R^2^ = 0.426). The type of RPD had a large effect size on chewing function (F (2,79) = 16.31, *p* < 0.001, partial η^2^ = 0.292), indicating substantial differences in masticatory outcomes among treatment groups.

Adjusted means indicated that patients rehabilitated with C-RPDs reported significantly higher CFQ scores (mean = 17.00; 95% CI: 15.71–18.30), reflecting poorer chewing function, compared with those treated with A-RPDs (mean = 12.40; 95% CI: 10.66–14.14) and I-RPDs (mean = 12.03; 95% CI: 10.70–13.36). Pairwise comparisons confirmed that C-RPD outcomes were significantly worse than both A-RPD (*p* < 0.001; mean difference = 4.60) and I-RPD (*p* < 0.001; mean difference = 4.97). No significant difference was observed between A-RPD and I-RPD groups (*p* = 1.000, mean difference = 0.37).

Baseline CFQ scores were also a significant predictor of post-treatment outcomes (F (1,79) = 15.17, *p* < 0.001, partial η^2^ = 0.161), indicating that individuals with poorer chewing function at baseline tended to maintain lower functional performance post-treatment. Education demonstrated a moderate influence (F (1,79) = 6.50, *p* = 0.013, partial η^2^ = 0.076), with higher education levels associated with slightly lower CFQ scores (better chewing function). Age, gender, and gender–treatment interactions were not significant predictors (*p* > 0.05).

A repeated-measures ANCOVA revealed a significant main effect of time on CFQ scores (F = 7.10, *p* = 0.001, partial η^2^ = 0.082), indicating that chewing function changed significantly across the three follow-up points (post-treatment, 1-year, and 5-year). Mean CFQ scores decreased slightly from post-treatment (13.84) to 1 year (13.36), followed by a marked increase at 5 years (19.22), reflecting a progressive decline in masticatory performance over time. Pairwise comparisons confirmed significant differences between post-treatment and 5-year follow-up (*p* < 0.001; mean difference = 5.37) and between 1-year and 5-year follow-up (*p* < 0.001; mean difference = 5.86).

The type of maxillary RPD had a highly significant effect on chewing function (F = 44.75, *p* < 0.001, partial η^2^ = 0.528). Patients rehabilitated with C-RPDs consistently exhibited the poorest chewing function (mean = 20.43; 95% CI: 19.20–21.66), whereas those treated with A-RPDs (mean = 13.59; 95% CI: 11.93–15.25) and I-RPDs (mean = 12.40; 95% CI: 11.14–13.66) reported significantly better outcomes. Bonferroni-adjusted comparisons confirmed that CFQ scores were significantly higher in the C-RPD group compared with both A-RPD (*p* < 0.001; mean difference = 6.84) and I-RPD (*p* < 0.001; mean difference = 8.03). No significant difference was observed between A-RPD and I-RPD groups (*p* = 0.762).

**Figure 3:**
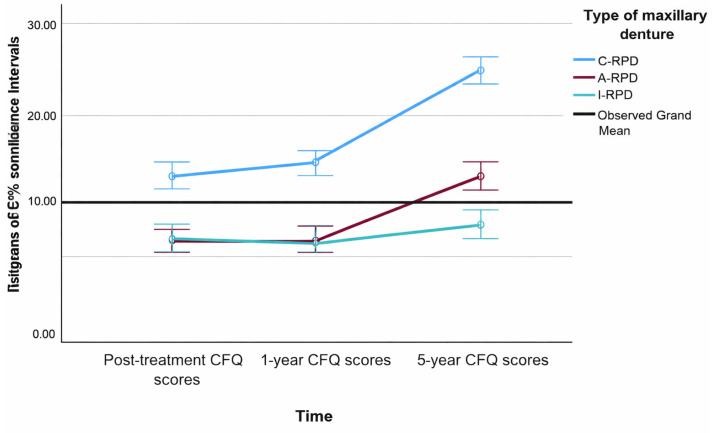
Estimated marginal means with 95% Confidence Intervals of the CFQ summary scores over time by types of maxillary RPDs.

The interaction between time and denture type was also significant (F = 41.75, *p* < 0.001, partial η^2^ = 0.511), indicating that the trajectory of chewing function over time differed substantially depending on the type of maxillary RPD. Patients rehabilitated with C-RPDs demonstrated the most pronounced deterioration over the five-year period, whereas those with attachment-retained and implant-retained designs maintained more stable chewing performance. Small but significant effects were observed for age (F = 4.33, *p* = 0.041, partial η^2^ = 0.051) and education (F = 6.94, *p* = 0.010, partial η^2^ = 0.080), while gender did not significantly influence CFQ outcomes (*p* > 0.05). Estimated marginal means with 95% confidence intervals for CFQ scores over time by treatment group are presented in Figure 3.

### 3.4. Clinical Maintenance During Follow-Up

Throughout the five-year follow-up period, all prostheses were evaluated at scheduled recall appointments. In the A-RPD and I-RPD groups, retention components (matrices and O-rings) were replaced when a reduction in retention was clinically detected or when patients reported loosening of the denture. In the C-RPD group, wrought wire clasps were regularly adjusted to improve retention, and fractured clasps were replaced when necessary. In all treatment groups, relining of maxillary and/or mandibular RPDs was performed when indicated to restore optimal fit and ensure comfort.

## 4. Discussion

Patients in this cohort indicated that the type of maxillary RPD had a substantial influence on esthetics, chewing function, and oral health-related quality of life. After adjustment for key confounders, both attachment-retained and mini-implant-retained RPDs consistently yielded superior patient-reported outcomes compared with clasp-retained RPDs immediately after treatment and throughout the five-year follow-up period.

Baseline conditions were comparable among groups due to strict inclusion criteria, which ensured similar anterior tooth support and comparable posterior ridge morphology in all participants. The antagonistic (mandibular) arch status did not introduce systematic bias, as all patients exhibited Kennedy Class I partial edentulism and used conventional mandibular RPDs. Therefore, adjustment for this factor in the statistical model was not required. It is also consistent with our previous 5-year follow-up study on MDI retained RPDs, which demonstrated no significant influence of opposing arch status or different loading protocols on long-term esthetic, functional, and clinical outcomes, or OHRQoL [6,42,50]. Thus, it is unlikely that variation in loading timing within the I-RPD group affected the present results.

To ensure comparable baseline conditions, identical inclusion criteria were applied across the treatment groups (C-RPD, A-RPD, and I-RPD), such as sufficient alveolar ridge dimensions. According to the Cawood and Howell classification [49], all exhibited posterior maxillary ridge forms within the III–IV range; however, none could be distinctly characterized as fully Class III or Class IV. Specifically, ridge width was not sufficient for placement of standard size implants, which would be expected in a typical Class III ridge, while ridge morphology was not fully knife-edged, as seen in Class IV. Additionally, ridge height in the molar region was <10 mm in all cases due to maxillary sinus pneumatization. Therefore, the Cawood and Howell classification was used as a general descriptive framework rather than a strict categorization tool, as individual ridge morphology in these patients represented transitional characteristics between Classes III and IV.

To further standardize baseline functional conditions, all dentures were fabricated using a lingualized occlusal scheme, without attempting bilateral balanced occlusion. Canine guidance was applied whenever adequate periodontal support and crown morphology of the canines allowed; when this was not feasible due to anatomical or restorative limitations, group function was established instead. Standardizing mandibular prosthetic conditions in this manner helped minimize the potential confounding influence of the opposing arch or occlusal scheme on chewing efficiency and OHRQoL outcomes.

Although the final sample in the I-RPD group consisted of 27 participants, the power analysis had already accounted for an anticipated attrition of up to 30% over the five-year follow-up period. Therefore, the reduction of one participant relative to the target baseline sample falls within the expected range and does not materially affect the statistical power of the study. Moreover, comparison of baseline characteristics between participants who completed the study and those who discontinued (age and sex distribution) showed no significant differences, indicating that attrition was unlikely to introduce systematic bias into the results.

Since dental patients generally seek treatment to restore function, improve comfort, and enhance esthetic appearance [56], it is important to provide clear and realistic information about expected treatment outcomes for different therapeutic options [11,17,56,57,58,59,60,61,62,63,64] over the long term. Beyond relieving pain and improving masticatory function, the esthetic outcome of RPD therapy plays a crucial role in overall satisfaction [6,8,9,11,14,65,66,67,68,69,70,71,72,73], particularly when the prosthesis or its components are located within the visible esthetic zone. Therefore, the chosen restoration should not compromise esthetic outcomes [11,17,18,22,25,28,30,31,33,44,62,63,64,74]. In clinical practice, dentists may face challenges when selecting the most appropriate long-term prosthetic solution, especially when several treatment options are viable. This decision becomes even more complex for patients with anatomical, medical, or financial limitations. In such cases, clinicians must also consider the psychosocial impact of impaired function and orofacial esthetics on the patient’s overall well-being.

The maxillary anterior region is the most visible during smiling and speech [75,76,77], therefore esthetic considerations are critical when planning a maxillary RPD. While C-RPDs are simple and cost-effective, the visibility of clasps on anterior teeth can compromise esthetics [17,40,41,78].

In this study, all three treatment modalities produced initial esthetic improvements; however, the C-RPD group showed the smallest gain, followed by a steady decline that returned to pre-treatment levels by the five-year follow-up. In contrast, both A-RPDs and I-RPDs yielded substantially greater immediate improvements in OES scores, followed by only a moderate decline over five years, with scores consistently remaining well above pre-treatment levels. Overall, C-RPDs resulted in the lowest patient satisfaction with orofacial esthetics, whereas A-RPD and I-RPD designs achieved significantly higher and comparable long-term outcomes. These findings underline the critical role of prosthesis design in maintaining esthetic results and support A-RPDs and I-RPDs as superior esthetic alternatives to conventional C-RPDs, aligning with previous research of negative influence of visible clasps on facial esthetics and patient satisfaction [14,17]. The presence of clasps in C-RPDs compromises smile appearance and is often a decisive factor in treatment acceptance [23,29]. In contrast, attachment-retained and implant-assisted RPD designs, which eliminate or reduce visible metal components, provide a more harmonious integration with the natural dentition and have consistently demonstrated superior esthetic outcomes [6,40,41,42]. However, esthetic satisfaction is not static but tends to decline over time for most prosthetic restorations, including complete dentures [4,8,21], despite initially high post-treatment scores. Esthetic outcomes evolve under the influence of clinical factors, such as material wear, staining of artificial teeth and acrylic resin bases due to pigments in food, drinks, smoking, or microbial colonization and anatomical changes including soft-tissue remodeling and alveolar ridge resorption [4,78,79,80,81,82,83,84]. Additionally, patient-related aspects such as shifting expectations and psychosocial adaptation also contribute to changes in esthetic perception over time [85,86,87,88]. These combined factors likely contributed to a 5-year decrease in OES scores across all groups, however with the most pronounced decline in the C-RPD group.

Prosthesis design also had a strong influence on OHRQoL and masticatory function. The OHIP-14 was selected for assessment of OHRQoL due to its extensive validation, international comparability, and proven reliability in Croatian populations [53]. Immediately after treatment, the highest OHIP-14 scores in the C-RPD group indicated great oral health-related difficulties, whereas those treated with A- and I-RPDs experienced significantly better outcomes. Strong association of pre-treatment and post-treatment scores may reflect stable individual psychosocial characteristics influencing how patients perceive oral conditions. Slightly higher OHIP-14 scores among older participants may be related to increased residual ridge atrophy and greater challenges in neuromuscular adaptation to removable prostheses with age [4,89,90,91,92].

While OHRQoL declined overtime in all groups, the deterioration was most pronounced in the C-RPD group (OHIP-14 scores increased from 17.6 post-treatment to 28.4 at five years, exceeding baseline levels). In contrast, both A-RPDs and I-RPDs maintained substantially lower scores throughout the observation period indicating more stable long-term OHRQoL. The I-RPD group demonstrated a modest trend toward more favorable 5-year outcomes than A-RPD in both OHIP-14 and CFQ measures, suggesting potential clinical relevance. These findings are consistent with previous research showing that conventional C-RPDs are associated with lower satisfaction and poorer self-perceived oral health compared with attachment or implant-assisted designs [4,6,8,9,26,31,32]. Systematic reviews and longitudinal studies have similarly reported that I-RPDs reduce functional limitations and psychosocial discomfort maintaining superior OHRQoL outcomes even after several years of use, in contrast to C-RPDs where satisfaction and function commonly declined over time [6,27,42,43,44,93,94,95,96,97,98].

In this study, the type of maxillary RPD had also a major influence on chewing function. Patients rehabilitated with C-RPDs consistently reported the poorest masticatory performance, whereas those with A-RPDs and I-RPDs achieved significantly better post-treatment outcomes, with no significant difference observed between the latter two groups., Baseline CFQ scores were a strong predictor of post-treatment scores, indicating that individuals who perceived greater functional limitations prior to treatment tended to report poorer chewing outcomes afterward as well. This pattern suggests again that stable individual response tendencies and psychological or behavioral factors may contribute to how patients evaluate their functional performance over time. Longitudinal changes further underscored the impact of prosthesis design. After one year, CFQ scores slightly decreased in the A-RPD and I-RPD groups, reflecting improved neuromuscular adaptation and functional efficiency compared to shorter post-treatment period. In contrast, the C-RPD group demonstrated an early increase in CFQ scores, indicating a decline in chewing performance. Over the five-year period, chewing function progressively deteriorated across all treatment groups, but this decline was most pronounced in the C-RPD cohort, while both A-RPDs and I-RPDs maintained considerably better and more stable masticatory function. The I-RPD group showed a modest trend towards the most favorable long-term outcomes. Findings generally indicate better masticatory adaptation in the A-RPD and I-RPD groups during the five years of function (Figure 2 and Figure 3). In the C-RPD group, CFQ scores eventually surpassed baseline levels, indicating deteriorating chewing function, likely due to progressive alveolar ridge resorption and reduced denture stability over time. Enhanced functional performance of I-RPDs may reflect the added retention and support from mini-implants, which transform primarily linear support into a more biomechanically favorable tripod or rectangular configuration, similar to designs with standard-width implants [93,94,95,96,97,98,99]. Our results are consistent with systematic reviews reporting superior masticatory performance in implant-assisted RPDs [27,44], and with clinical studies evaluating mini-implant-retained RPDs [6,100,101,102,103,104,105]. One study even demonstrated reduced mobility of remaining abutment teeth when mini-implants were incorporated under existing RPDs over a three-year period [103].

One multicenter randomized clinical trial reported earlier improvements in patient satisfaction when mini-implants were loaded immediately [105]. This study included early (6–8 weeks) or delayed (3–4 months) loading, both requiring a brief delay before full mastication. Consistent with previous research [6], which found no significant differences in clinical or patient-reported outcomes between early and delayed loading of MDI-retained RPDs, the timing of loading likely did not influence the present results. Nevertheless, future research with larger sample sizes and subgroup-based analyses is recommended to further evaluate the potential effect of loading protocol on long-term outcomes.

Patient-reported outcomes aligned with clinical findings over the five-year follow-up. Periodontal health of anterior abutments remained stable, and no mini-implant failures occurred in the I-RPD group, yielding a 100% survival rate. Retentive components were replaced and dentures relined as needed, with maintenance routines similar across groups. Overall, the superior outcomes in the A-RPD and I-RPD groups were supported by stable prosthesis function and soft-tissue health, confirming the long-term reliability of these designs.

Potential confounders were addressed by adjusting for baseline scores when evaluating treatment effects and longitudinal changes in OES, OHIP-14, and CFQ. Baseline scores consistently influenced follow-up results, reflecting stable individual assessments over time. Age had minimal impact on post-treatment OHIP-14 scores, while higher education was modestly associated with fewer reported chewing difficulties indicating that psychosocial and behavioral factors, such as greater understanding of prosthesis limitations among more educated individuals, may influence patient-reported outcomes and perceived satisfaction. 

Treatment cost influences prosthesis selection. While A-RPDs and I-RPDs require higher initial investment than C-RPDs, their superior retention, esthetics, and long-term stability may reduce adjustments and early replacement, making them cost-effective over time. However, in this study, mini-implant costs did not affect treatment choice, as implant placement was fully funded, so financial considerations did not confound allocation in the I-RPD group. Results of the present study may support clinicians in shared decision-making by aligning treatment recommendations with patient preferences, anatomical conditions, and financial considerations.

Overall, conventional C-RPDs demonstrated important limitations with a progressive decline in all outcomes over time. A- and I-RPD designs provided substantially better initial improvements and demonstrated greater long-term stability across all patient-reported measures in this cohort, emphasizing the fundamental influence of prosthesis design on d-PROMs. Clinically, this highlights the importance of discussing both immediate outcomes and long-term performance when presenting treatment options. Evidence-based communication can help dentists guide patients toward choices that align with their functional needs, esthetic expectations, anatomical limitations, and budget.

This study has several limitations that should be acknowledged. First, the non-randomized design may have introduced allocation bias, as participants selected the treatment modality. However, this reflects real-world clinical practice, where treatment choice is influenced by patient preferences, expectations, and financial considerations. Thus, although this design limits internal comparability between groups, it enhances the external relevance of the findings to routine prosthodontic decision-making. Second, the self-reported nature of d-PROMs may be influenced by individual psychological factors, expectations, or motivation. Nevertheless, the use of validated questionnaires and adjustments for baseline values helped mitigate this potential bias. Third, although all prostheses were periodically reviewed and maintained during the 5-year follow-up. Differences in the frequency and timing of maintenance procedures, such as O-ring or matrix replacement, clasp activation, or relining, may have influenced masticatory efficiency and patient satisfaction at specific time points. The study’s single-center design and relatively limited sample size may restrict the generalizability of the findings to broader patient populations.

Future research should involve larger, more diverse patient groups, ideally through multicenter, randomized longitudinal studies. Combining d-PROMs with objective measures of denture performance and cost-effectiveness would improve evidence-based guidelines. Studies should also examine how function and esthetics independently affect oral health-related quality of life, as these factors may differently influence patient satisfaction and treatment success.

## 5. Conclusions

Conventional C-RPDs were associated with lower OHRQoL, poorer esthetic satisfaction, and chewing function declining below baseline over five years. In contrast, A-RPDs and mini-implant-RPDs showed better, more stable outcomes, with I-RPDs performing best long-term. Within the limitations of this observational study, A-RPDs and I-RPDs appear more functionally effective and esthetically favorable than clasp-retained designs when clinically feasible.

## Figures and Tables

**Table 1:** Pre-treatment, post-treatment, 1-year, and 5-year follow-up summary scores for: (**a**) Orofacial Esthetic Scale (OES), (**b**) Oral Health Impact Profile-14 (OHIP-14), and (**c**) Chewing Function Questionnaire (CFQ), according to the type of maxillary RPD.

a.						
Orofacial Esthetic Scale (OES; 8–40)						
Time Point	Treatment Group	*N*	Mean	SD	Min	Max
Pre-treatment	C-RPD	30	23.00	4.32	15.00	30.00
	A-RPD	31	22.10	4.64	10.00	33.00
	I-RPD	27	21.41	3.19	16.00	28.00
Post-treatment	C-RPD	30	30.33	3.21	25.00	39.00
	A-RPD	31	36.45	3.01	29.00	40.00
	I-RPD	27	36.56	1.97	32.00	39.00
1-year follow-up	C-RPD	30	28.23	3.95	18.00	35.00
	A-RPD	31	34.90	2.83	28.00	40.00
	I-RPD	27	34.89	1.60	32.00	37.00
5-year follow-up	C-RPD	30	25.57	4.48	19.00	34.00
	A-RPD	31	30.52	3.58	20.00	37.00
	I-RPD	27	32.41	1.82	29.00	35.00
b.						
Oral Health Impact Profile-14 (OHIP-14; 0–56)						
Time point	Treatment group	*N*	Mean	SD	Min	Max
Pre-treatment	C-RPD	30	28.23	6.43	16.00	50.00
	A-RPD	31	27.00	7.74	9.00	42.00
	I-RPD	27	29.11	5.05	19.00	38.00
Post- treatment	C-RPD	30	17.63	3.85	11.00	26.00
	A-RPD	31	10.84	3.51	5.00	21.00
	I-RPD	27	10.85	2.68	6.00	17.00
1-year follow-up	C-RPD	30	18.80	4.63	11.00	28.00
	A-RPD	31	11.45	3.32	6.00	20.00
	I-RPD	27	11.33	2.75	7.00	19.00
5-year follow-up	C-RPD	30	28.43	5.95	18.00	48.00
	A-RPD	31	17.35	5.03	2.00	28.00
	I-RPD	27	13.11	2.29	9.00	18.00
c.						
Chewing Function Questionnaire (CFQ; 0–40)						
Time point	Treatment group	*N*	Mean	SD	Min	Max
Pre-treatment	C-RPD	30	24.83	3.94	19.00	33.00
	A-RPD	31	25.52	7.68	9.00	38.00
	I-RPD	27	26.93	3.46	19.00	33.00
Post- treatment	C-RPD	30	16.43	3.49	10.00	25.00
	A-RPD	31	11.61	3.67	6.00	23.00
	I-RPD	27	12.04	3.90	5.00	20.00
1-year follow-up	C-RPD	30	16.90	3.95	9.00	24.00
	A-RPD	31	11.29	2.88	6.00	19.00
	I-RPD	27	11.04	3.04	5.00	18.00
5-year follow-up	C-RPD	30	25.80	3.90	18.00	34.00
	A-RPD	31	16.97	3.93	9.00	26.00
	I-RPD	27	13.44	2.86	7.00	20.00

C-RPD = clasp retained RPD; A-RPD = precision attachment retained RPD; I-RPD = mini-implant retained RPD; *N* = number; Min = minimum; Max = maximum.

## Data Availability

The original contributions presented in this study are included in the article. Further inquiries can be directed to the corresponding author.

## Abbreviations

The following abbreviations are used in this manuscript:

OES	Orofacial Esthetic Scale
OHRQoL	Oral Health Related Quality of Life
OHIP-14	Oral Health Impact Profile
C-RPD	Clasp-retained removable partial denture
A-RPD	Attachment-retained removable partial denture
I-RPD	Implant retained removable partial denture
d-PROMs	Dental patient-reported outcome measures
CFQ	Chewing Function Questionnaire
